# A Novel Crosslinking Method for Improving the Anti-Calcification Ability and Extracellular Matrix Stability in Transcatheter Heart Valves

**DOI:** 10.3389/fbioe.2022.909771

**Published:** 2022-07-12

**Authors:** Xiaoke Qi, Zhenlin Jiang, Mingzhe Song, Zhenjie Tang, Xinlong Xie, Yuhong Liu, Qiying Wu, Zhongshi Wu

**Affiliations:** Department of Cardiovascular Surgery, The Second Xiangya Hospital, Central South University, Changsha, China

**Keywords:** ribose, elastin, calcification, durability, biomaterial

## Abstract

More than 200,000 patients with aortic diseases worldwide undergo surgical valve replacement each year, and transcatheter heart valves (THV) have been more widely used than ever before. However, THV made by the glutaraldehyde (Glut) crosslinking method has the disadvantage of being prone to calcification, which significantly reduces the durability of biomaterials. In this study, we applied a novel crosslinking method using ribose in THV for the first time, which can decrease calcification and increase the stability of the extracellular matrix (ECM). We incubated the bovine pericardium (BP) in ribose solution at 37°C by shaking for 12 days and confirmed that the structure of the BP was more compact than that of the Glut group. Moreover, the ribose method remarkably enhanced the biomechanical properties and provided reliable resistance to enzymatic degradation and satisfactory cellular compatibility in THV. When the BP was implanted subcutaneously *in vivo*, we demonstrated that ECM components were preserved more completely, especially in elastin, and the immune-inflammatory response was more moderate than that in the Glut treatment group. Finally, the ribose-cross-linked materials showed better anti-calcification potential and improved durability of THV than Glut-cross-linked materials.

## Introduction

Valve replacement surgery is the first choice for patients with heart valve diseases (Brown et al., 2009). Every year, more than 200,000 aortic valve replacement surgeries occur globally, and the use of transcatheter heart valves (THV) has increased significantly in the past 20 years (Bui et al., 2021). With the popularization of transcatheter aortic valve replacement (TAVR) technology, a THV exhibits excellent hemodynamic characteristics and provides a safe and minimally invasive means (Paradis et al., 2015). At present, the material of bioprostheses used in clinical operations is derived from the bovine pericardium (BP) or porcine pericardium, and both of them are treated by the chemical crosslinking method of glutaraldehyde (Glut) (Manji et al., 2014). However, TAVR valves face similar material failure mechanisms as surgical aortic valve replacement (SAVR) valves, calcification, and valvular degeneration of the biomaterials are the main causes. Furthermore, transcatheter delivery requires THV to be crimped before deployment, as the valves often undergo additional mechanical stresses which would reduce the durability of THV. (Schoen and Levy, 2005; Rodriguez-Gabella et al., 2017; Bui et al., 2021).

In contrast to previous simple views on time/age-related degenerative processes, calcification is now generally considered an active process that is cell-mediated, multi-factor influenced, and an immune-inflammatory response (Simionescu, 2004; Rodriguez-Gabella et al., 2017). The biomaterial is gradually infiltrated by inflammatory cells, and it secretes calcium-binding protein, releases oxygen free radicals, and oxidative stress damage leads to atherosclerotic-like characteristic lipid deposition and the formation of new blood vessels (Mylotte et al., 2015). The THV exhibits extracellular matrix (ECM) degradation and degeneration driven by mechanical stress (Li et al., 2019). Collagen and elastin fibers were injured during circulation. Finally, free calcium ions and phosphate are combined and deposited in the specific site of the damaged ECM in the THV, which is treated by the Glut crosslinking method and implanted in the body (Tam et al., 2015; Lindman et al., 2016). In the hole zone, calcium phosphate-hydroxyapatite (HAP) crystals are formed and gradually expand, making the valve leaflets stiff, and limiting mobility, resulting in valve dysfunction, and significantly reducing durability (Shah and Vyavahare, 2008).

Therefore, methods to reduce calcification in the THV have been widely studied, for instance, to remove cell components and reduce the immunogenicity of biomaterials while protecting the composition and three-dimensional ultrastructure of the ECM as much as possible (Crapo et al., 2011). Improving the stability of collagen and elastin is essential for maintaining the morphology and function of the valve (Monte-Nieto et al., 2020). Adding effective hyaluronidase inhibitors or natural glycosaminoglycans (GAGs) decreases mineral deposition (Tam et al., 2015). There are several alternative crosslinking technologies involving dye-mediated photo fixation, carbodiimide, genipin, and radical polymerization-crosslinking methods (Baird et al., 2016; Tam et al., 2017; Guo et al., 2018). Although all of these methods have been reported to have the effect of reducing calcification, they have not yet been widely applied in clinics. The research direction is to find a low-toxicity or nontoxic method that can form stable covalent bond crosslinking, preserving collagen and elastin at the same time (Zilla et al., 2008; Li et al., 2019).

Ribose (Rib), as one of the constituent components of RNA, has strong reducing properties. Rib serves as a new type of biological crosslinking agent that has been proven to crosslink the amino groups of collagen (Krishnakumar et al., 2017a). It is reported to be used in the production procedure of collagen scaffolds to fill bone tissue and promote the repair and regeneration of bone defects (Willett et al., 2015). The main component of connective tissue in the BP is collagen with a triple helix structure, which constitutes the reticular framework in valves and provides the main stress support (Monte-Nieto et al., 2020). Therefore, we assume that the Rib crosslinking method can be applied in the preparation of the BP to improve the stability of the ECM and reduce the thickness of the biomaterial, which is beneficial to be crimped before transcatheter delivery. The immunogenicity of the heterogeneous tissue is removed by decellularization. When the crosslinking process is completed, BP biomaterials are evaluated for their anti-calcification ability *in vitro* and *in vivo*.

## Materials and Methods

### Materials

Glut (OHC(CH_2_)_3_CHO, purity 50 wt%) was purchased from Aladdin (Shanghai, China). Ribose (C_5_H_10_O_5_, purity 99 wt%), collagenase (type I), and Triton X-100 were purchased from Sigma Aldrich (USA). Elastase was purchased from Shanghai Macklin Biochemical Co., Ltd. (Shanghai, China).

### Crosslinking of the Transcatheter Heart Valve

Fresh BP was harvested from the local slaughterhouse, the warm ischemia time was controlled within 30 min, soaked in 0.9% saline and ice, and transported to the laboratory. First, the fat and connective tissue on the surface of the BP were removed, cut into an 8 × 6 cm^2^ rectangle for use, and then washed with 0.9% saline three times for 10 min each.

The BP was decellularized using the following method (Crapo et al., 2011): first, fresh BP was incubated with 0.25% Triton in phosphate-buffered saline (PBS) and shaken for 24 h. The solution was decanted, and the biomaterial was then treated with DNase (3 U/ml) and RNase (0.03 mg/ml) solutions containing MgCl_2_ (2.5 mM) and CaCl_2_ (0.1 mM) and stirred at 37°C for 24 h. The solution was decanted, and the BP was rinsed with distilled water for 24 h at room temperature. Finally, the BP was rinsed with PBS three times for 5 min each time. Small pieces of the BP were freeze-dried and weighed for DNA quantification (*n* = 4). A DNA detection kit (TIANamp Genomic DNA kit) was used to extract the total cellular DNA in the BP tissue, which was quantified by a NanoDrop 2000 (Thermo Fisher Scientific, MA, United States) and then normalized to the dry weight of the BP.

**FIGURE 1:**
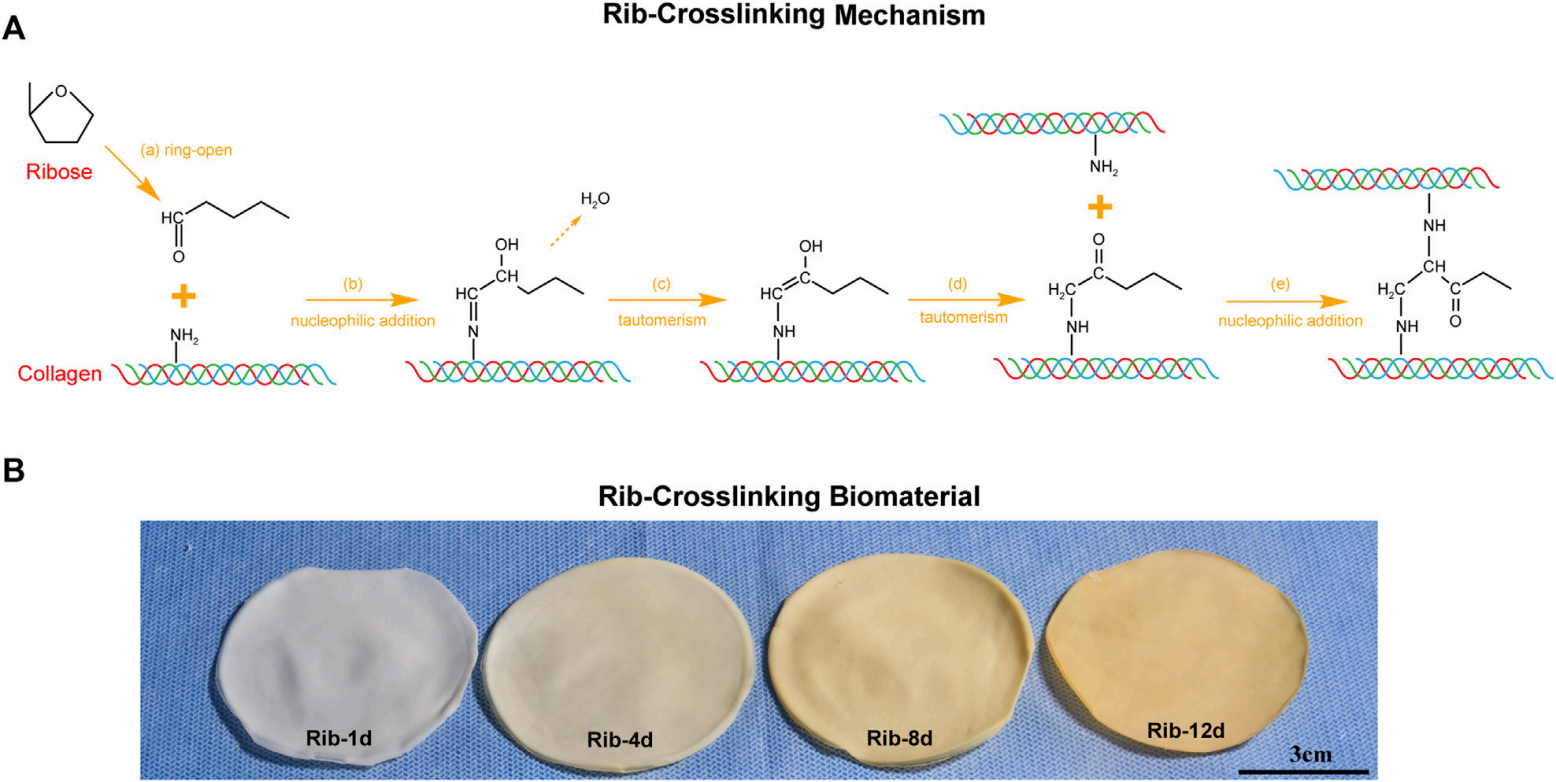
Rib crosslinking method of the bovine pericardium (BP). **(A)** Schematic illustrations of the mechanism by which Rib stabilizes collagen in the ECM. **(B)** Effect of Rib crosslinking time on the macroscopic appearance of biomaterials. Scale bars: 3 cm.

Two different crosslinking methods are used to process the decellularized BP: (1) Glut control group and (2) Rib new technology. Figure 1 shows the fundamental mechanism of the Rib methods. The detailed crosslinking protocol is described as follows:

(1) GLUT——Fresh BP was treated with 0.6% Glut in 50 mM 4-(2-hydroxyethyl)-1-piperazineethanesulfonic acid (HEPES)-buffered saline (pH 7.4) at room temperature with shaking for 1 day. The solution was decanted, and the BP was stored with 0.2% Glut in 50 mM HEPES buffered saline (pH 7.4) for 6 days.
(2) RIBOSE——Fresh BP was treated with 80 mM Rib in ethanol and PBS (50:50 vol%) solution (pH 7.4) at 37°C with shaking. The crosslinking reaction lasted for 1 day, 4 days, 8 days, and 12 days (the solution was refreshed every 4 days).

### Ninhydrin Assay

According to previous reports (Tam et al., 2015; Guo et al., 2018), the content of free amino groups in the BP was determined by the ninhydrin assay. The cross-linked BP tissue was cut into a size of 0.5 × 0.5 cm^2^. After washing three times with deionized water, it was frozen and dried to a constant weight. Each sample was placed in a centrifuge tube and 1 ml of 1% w/v ninhydrin was added. The centrifuge tube was transferred to water, heated, and then cooled in a boiling water bath for 20 min. The OD value of the supernatant was measured at 570 nm in a microplate reader, and a blank ninhydrin solution was used for background correction. Gradient concentrations of glycine were diluted to generate a standard curve.

### Nuclear Magnetic Resonance (^1^H-NMR)

The BP tissue was cut into a size of 0.5 × 0.5 cm^2^ (*n* = 3) and then rinsed three times with deionized water. The washed tissue was incubated with 6 N HCl at 50°C for 4 h, and the supernatant was extracted, frozen, and dried. Dry tissue powder was dissolved in D_2_O (50 mg/ml) and monitored using ^1^H-NMR analysis on a Bruker Avance III 400 MHz spectrometer (Switzerland).

### Scanning Electron Microscopy

The biomaterial specimens (*n* = 3) were rinsed with PBS solution three times, dehydrated with gradient alcohol, soaked in tertiary butanol for 2 h, and freeze-dried. A carbon vacuum was coated on the surface of each sample. The BP samples were examined (5 spots per sample) using a TESCAN MIRA4 LMH (Tescan, Czech Republic) scanning electron microscope at an accelerating voltage of 15 kV.

### Resistance to Enzymatic Treatment

The BP was rinsed with deionized water and cut into 2 × 2 cm^2^ pieces (*n* = 6). The water on the surface was absorbed by using filter paper and the wet weight was recorded as W_0_. Then, these tissues were frozen, lyophilized for 24 h, and weighed as W_1_. The BP patches were treated with 125 U/ml collagenase (type I) in 100 mM Tris (10 mM CaCl_2_, pH = 7.4) at 37°C for 24 h or 30 U/ml elastase (derived from porcine pancreatic) in 100 mM Tris (1 mM CaCl_2_, pH = 7.8) at 37°C for 48 h. Digested BP tissues were rinsed with deionized water three times, frozen, and lyophilized for 24 h again. The final weight was recorded as W_2_.

$$Weight\;Loss\%=\left(W_{1}{-W}_{2}\right)/W_{1}\text{,}\;Water\;Content\%=\left(W_{0}{-W}_{1}\right)/W_{0}.$$

Digested BP tissues were embedded in paraffin and sectioned at 5 µm for light microscopy analysis. Masson’s trichrome (MT) method was used for collagenase-digested BP, and the elastic van Gieson (EVG) method was used for elastase-digested BP.

### Mechanical Characterization

The tissue specimens (*n* = 12) were cut into 5 × 1 cm^2^ pieces, and the average thickness of the biomaterials was measured at three positions (head, mid, and tail). An electronic tensile testing machine (Instron, United States, electronic universal material testing machine) was used to perform uniaxial tensile testing (UTS) on the BP specimens at room temperature. The tensile rate was set to 50 mm/min. UTS was determined from the uniaxial data, defined by the peak stress and maximum deformation withstood by the BP specimens. Young’s modulus (MPa) was also quantified by means of the tangent modulus defined as the slope of the stress-strain curve in the high modulus linear region, which reflected the collagen fiber extension of the biomaterial.

### Thermal Stability Test

BP tissues (*n* = 6) were cut into 5 × 1 cm^2^ pieces and incubated in distilled water at room temperature. HG-1 leather shrinkage temperature tester (Sichuan Chengdu Dachengxing Digital System Co., Ltd. China) was used to heat the distilled water (2°C per minute). The thermal stability of the BP was recorded as the thermal shrinkage temperature (Td).

### Cytotoxicity

The human umbilical vein endothelial cell line EAhy926 was cultured in Dulbecco’s Modified Eagle Medium with 10% fetal bovine serum (DMEM/10% FBS).

All tissues were cut into 0.5 × 0.5 cm^2^ pieces (*n* = 3), sterilized by γ-rays to avoid bacterial growth, and rinsed with deionized water three times. The tissues were incubated in DMEM/10% FBS at 37°C for 24 h at a density of 2 ml/cm^2^. The media were replaced with a supernatant from the scaffold cultures diluted with DMEM/10% FBS at a ratio of 1:2. The cells were cultured for 1, 3, and 5 days at 37°C. Cell viability in the tissues was tested using a mitochondrial metabolic (MTT) assay. The optical density was determined using a microplate reader at 570 nm.

### EAhy926 Growth on the Biomaterial

The biomaterials were cut into 0.5 × 0.5 cm^2^ patches (*n* = 3), sterilized by γ-rays, and rinsed with deionized water three times to avoid bacterial growth. The patches were soaked in DMEM/10% FBS for 24 h and replaced in a 48-well plate. A total of 10 × 10^4^ сells in 20 μl were seeded on each patch. After 30 min, 500 μl of the medium was added to each well and was changed every 2 days. After culturing for 7 days, patches of the biomaterial were transferred to clean wells and washed with PBS solution. The dye mix (6-CDCFDA, 1:10) was added to each well and incubated for half an hour. Cells were visualized using a fluorescence microscope (Leica DMI 4000B).

### *In Vivo* Calcification Model

All tissues were cut into 1 × 1 cm^2^ pieces and rinsed with deionized water three times after fixation (*n* = 12). The cells were sterilized by γ-rays to avoid bacterial growth and remained in 60% ethanol solution until implantation. Four-week-old male Sprague–Dawley rats were anesthetized with pentobarbital sodium (0.1 mg/ml) via intraperitoneal injection. A longitudinal surgical incision was made on the back of rats, and four subdermal pockets were created on both sides of the incision. BP tissues were blotted dry and carefully implanted as flat as possible in each of the subcutaneous pockets. A 6-0 Prolene line was used to fix BP tissues and suture surgical incisions. After 90 days of implantation, the BP samples with fibrous capsules were harvested, and 1/2 of the sections were fixed with formalin for histological analysis. The other 1/2 sections were placed immediately on dry ice and frozen at −80°C for further examination.

All laboratory animals received humane care and were approved by the Second Xiangya Hospital of Central South University Animal Experiment Ethics Committee.

### Histology and Immunological Analysis

BP tissues were preserved in formalin, dehydrated, embedded in paraffin, cut into 5 μm sections, and analyzed by light microscopy. Hematoxylin and eosin staining was used to visualize the cellular response to implants and evaluate the thickness of the fibrous capsule. Masson’s trichrome (MT) staining was used to assess the ECM content, such as the distribution of collagen fibers. Victoria Blue staining was used to visualize elastin fibers. Von Kossa staining was used to visualize calcium deposition in the implanted biomaterial, and calcium deposits appeared black.

For immunohistochemical (IHC) staining and immunofluorescence (IF), sections were deparaffinized and rehydrated. The sections were incubated with primary antibodies at 4°C overnight after antigen retrieval. A rabbit anti-rat CD68 antibody (Servicebio Co., Ltd. Wuhan, China; dilution 1:500) was used to label macrophage cells (red). A rabbit anti-rat CD3 antibody (Servicebio Co., Ltd. Wuhan, China; dilution 1:500) was used to label T cells (red).

### Mineral Analyses

Half of the tissues harvested from the rat implantation model were frozen, lyophilized, weighed, and acid-hydrolyzed in 2 ml of 6 N HCl for 4 h at 50°C. The tissue solutions were centrifuged at high speed and the supernatants were diluted at 1:10 in deionized water. The calcium and phosphorous contents in the samples were analyzed using a Spectro Arcos ICP-7400 Spectrometer (Spectro Analytical Instruments, Thermo Fisher, USA) at Central South University Powder Metallurgy Laboratory. Dilution ratios were used to calculate the mineral content of the tissues, and values were normalized to dry sample weight.

### Statistical Analysis

The results are presented as the mean ± standard deviation (SD). A value of *p* < 0.05 was considered statistically significant. The data were analyzed for statistical significance by Student’s t-test for comparison of two groups and one-way ANOVA with the Bonferroni correction t-test for comparison of multiple groups.

## Results

### Crosslinking of the Biomaterial

The dependence of the degree of crosslinking on the soaking time is shown in Figure 1. This corresponds to the pigmentation which increased noticeably in intensity between 1, 4, 8, and 12 days. When the BP was soaked in Rib solution for 1 day, the biomaterial still showed a white color and no significant difference from the fresh BP. When the BP was soaked for 12 days, the biomaterial appeared yellow–brown.

**FIGURE 2:**
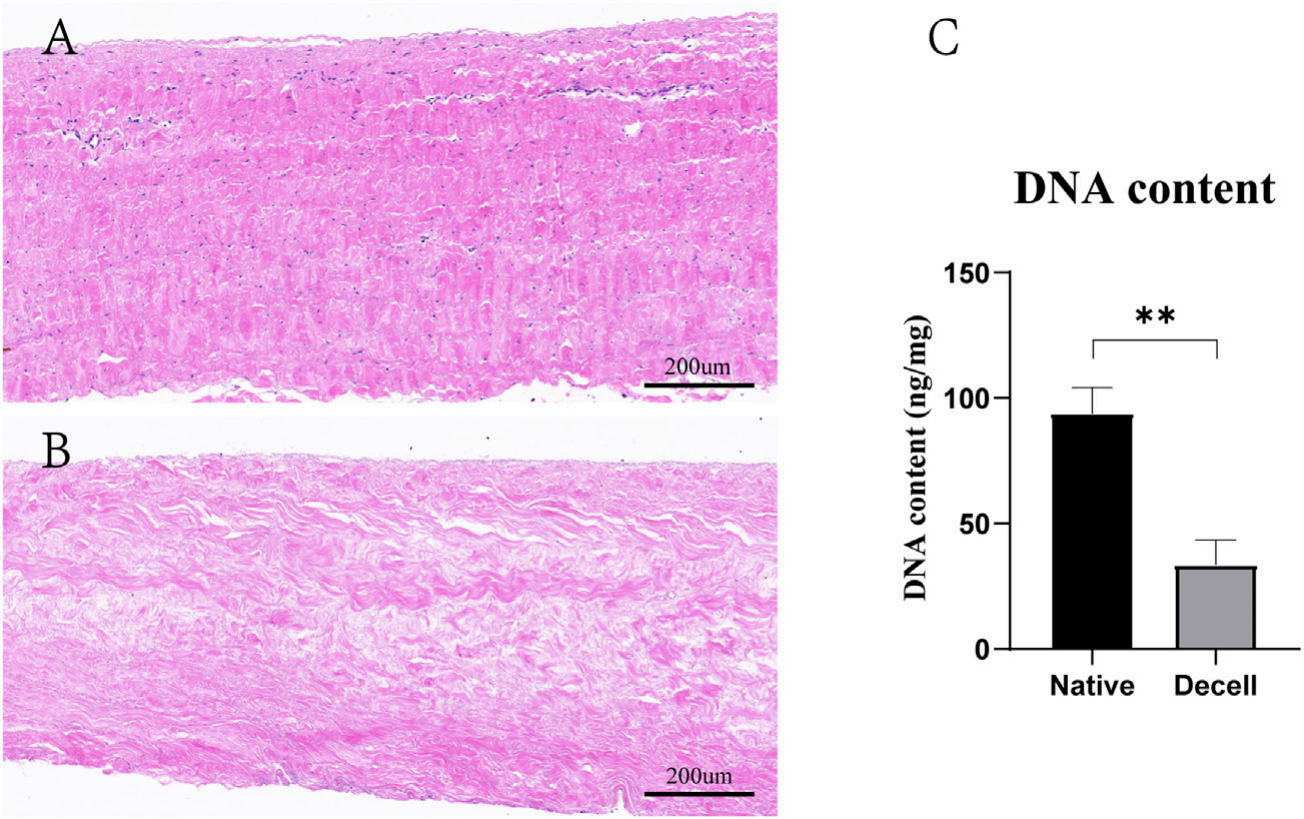
Decellularization process of the BP. **(A)** H&E staining of the native BP. **(B)** H&E staining of the decellularized BP. **(C)** Quantitative determination of the DNA content (*n* = 4) in the native BP and decellularized BP (scale bars: 200 µm; *p < 0.05, **p < 0.01, and ***p < 0.001; ns represents no significant difference).

According to H&E staining, we found that the nucleus and cytoplasm were completely removed after the decellularization procedure (Figures 2A,B). This was further confirmed by the significant difference in DNA material quantification. The DNA content in the fresh BP was 93.8 ± 10.4 ng/mg and 33.5 ± 9.9 ng/mg after the decellularization procedure (Figure 2C).

### Organic Structure on NMR and the Ninhydrin Reaction

**FIGURE 3:**
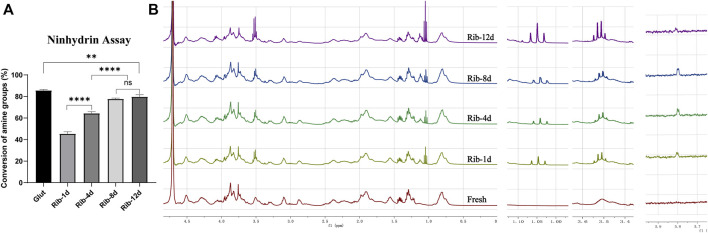
Changes in the reactive groups of the BP. **(A)** Ninhydrin assay to estimate the conversion of amine groups (*n* = 3) in the BP. **(B)** Representative ^1^H-NMR spectrum of the hydrolyzed product of the BP. Fresh BP, brown; Rib-1d, yellow; Rib-4d, green; Rib-8d, blue; Rib-12d, purple (*p < 0.05, **p < 0.01, and ***p < 0.001; ns represents no significant difference).

The amino group proved to be the main crosslinking site for Rib, and the ninhydrin reaction was used to detect the degree of crosslinking by analyzing the concentration of free amine groups. The amine conversion values of Rib-1d, Rib-4d, Rib-8d, and Rib-12d were 45.5 ± 1.9%, 64.3 ± 1.6%, 77.7 ± 0.8%, and 79.7 ± 1.8%, respectively (Figure 3A). The free amine groups decreased with Rib crosslinking time. The Glut crosslinking method had a conversion value of 85.6 ± 0.8% as a positive control.

^1^H-NMR spectra were used to monitor the conversion of Rib crosslinking. As shown in Figure 3B, the presence of a new signal (1.05 ppm) of enamine groups in the spectrum demonstrated that they were successfully introduced onto the Rib crosslinking BP. Signals corresponding to alkene groups were observed at 3.51 ppm in the spectrum of Rib crosslinking BP. The protons of the carbonyl group resonated as a doublet at 5.80 ppm. No signal of these functional groups was detected in the ^1^H-NMR spectra of the fresh BP. The NMR spectra indicated a possible conversion process of the Rib crosslinking method.

### Surface Structure on SEM

**FIGURE 4:**
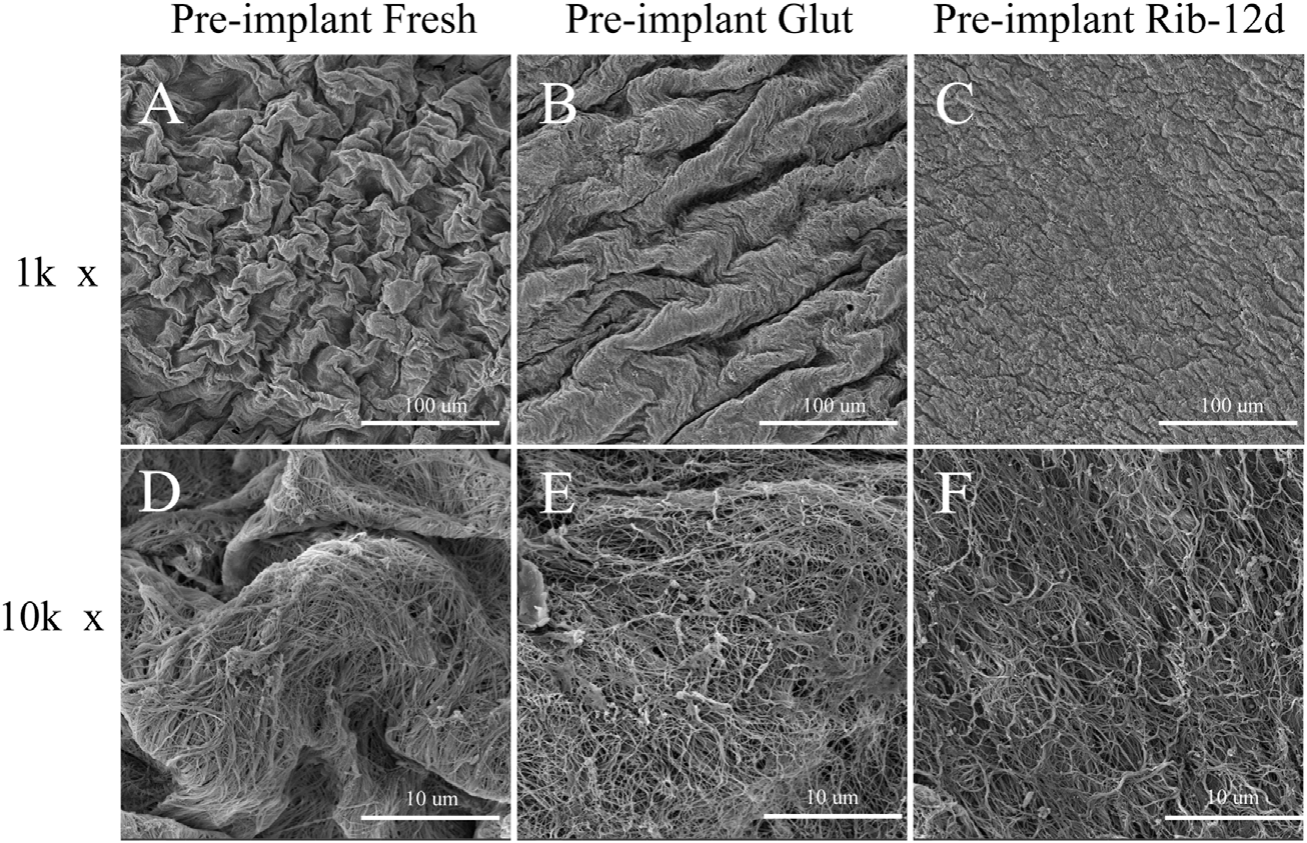
SEM images of BP surface structures before implantation. **(A,D)** Fresh BP. **(B,E)** Glut-treated BP. **(C,F)** Rib-treated BP. Scale bars: 100 μm **(A–C)** and 10 μm **(D–F)**.

**FIGURE 5:**
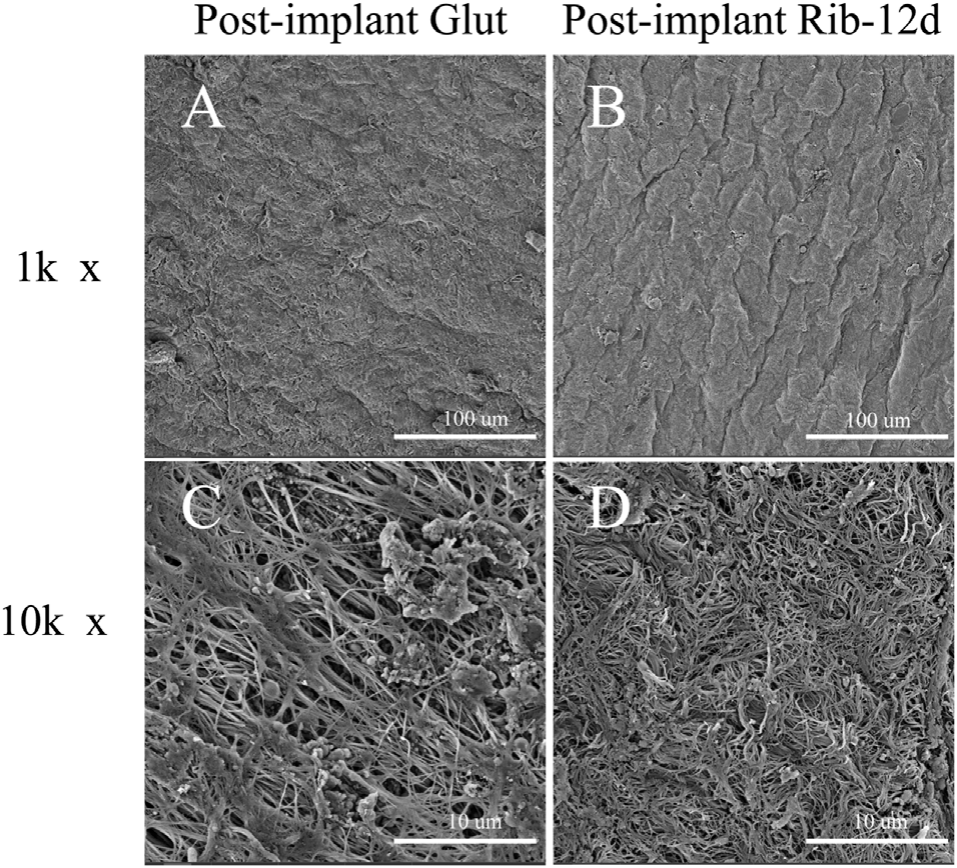
SEM images of BP surface structures after implantation. **(A,C)** Glut-treated BP was implanted into rats for 90 days. **(B,D)** Rib-treated BP was implanted into rats for 90 days. Scale bars: 100 μm **(A,B)** and 10 μm **(C,D)**.

SEM results showed that the surface structure of the BP in the fresh group was irregular and that the fiber arrangement was loose (Figures 4A,D). The BP in the Glut group had a regular and compact fiber structure, but the surface of the biomaterial was uneven and shrank obviously (Figures 4B,E). The BP in the Rib group had a smooth surface, compact structure, and regular fiber orientation and was connected to a network (Figures 4C,F). After implantation, the surface of the BP in the Rib group was flat, the structure was still compact, the fibers were clear, and there was no significant change compared to before implantation (Figures 5B,D). The BP structure in the Glut group was loose, a large amount of granular hyperplasia was obvious, and the fiber structure was destroyed (Figures 5A,C).

### Enzyme Stability Studies

Enzyme digestion assays were used to evaluate ECM stability. After the BP tissues were challenged with type I collagenase, all the cross-linked groups showed significantly lower weight loss than the fresh BP group (Figure 8A). However, the Rib group with short-time exhibited more weight loss (Rib-1d: 73.1 ± 3.2%, Rib-4d: 35.6 ± 4.8%) than the long-time group (Rib-12d:8.4 ± 1.7%); this data suggest that the long-time crosslinking method might provide more stable protection for collagen. In addition, the Rib-12d group showed no significant difference from the Glut group (5.3 ± 1.2%), indicating that collagen within the BP biomaterial was sufficiently protected in both crosslinking methods.

**FIGURE 6:**
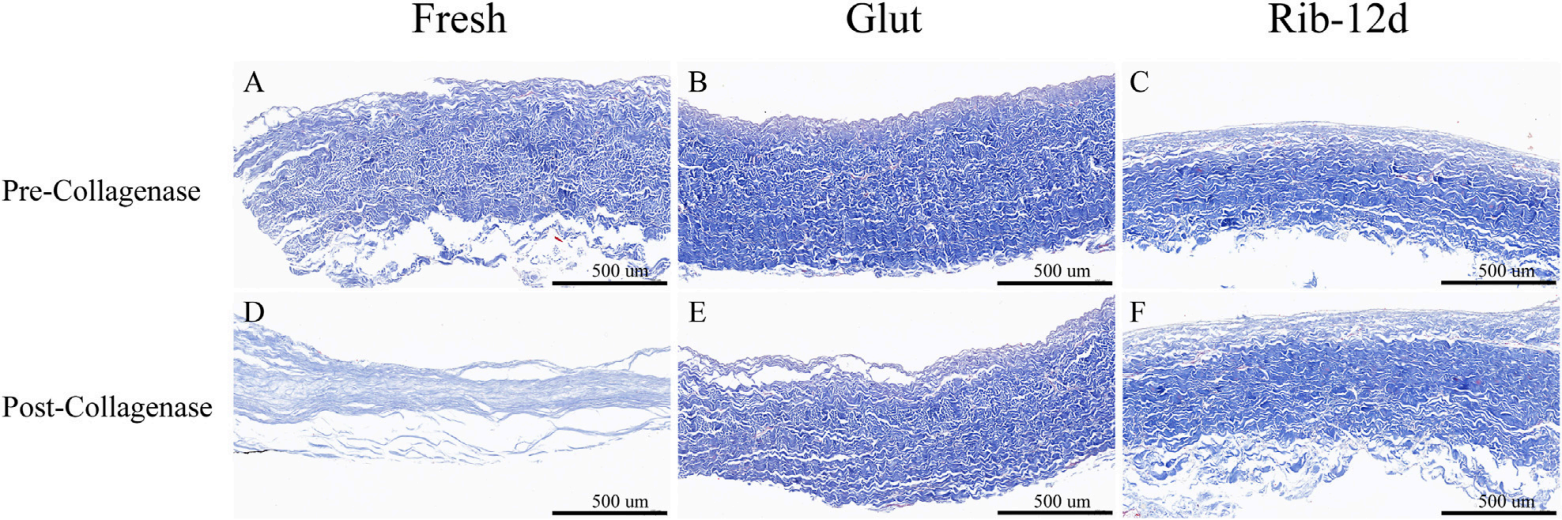
Resistance to the collagenase enzymatic challenge of the BP with Masson’s trichrome (MT) staining. **(A,D)** Fresh BP. **(B,E)** Glut-treated BP. **(C,F)** Rib-treated BP. Collagen are stained blue. Scale bars: 500 μm **(A–F)**.

BP tissues digested by collagenase were embedded in paraffin again and stained with Masson’s trichrome to observe the collagen morphological structure. After the challenge with collagenase, the BP structure of the fresh group was loose, almost lost its normal shape, and collagen fibers were obviously degraded and digested (Figure 6D). The structures of the Glut and Rib-12d groups were still compact (Figures 6E,F), and the collagen fibers stained blue and did not change compared with the pre-collagenase state (Figures 6B,C).

When BP tissues were challenged with elastase for 48 h (Figure 8B), weight loss in the Glut group (10.6 ± 1.4%) and fresh group (15.6 ± 2.0%) was different from that in the Rib-1d group (6.9 ± 0.9%) (*p* = 0.017). Furthermore, there were no significant differences between the Rib-1d group, Rib-4d group (5.6 ± 1.5%), Rib-8d group (6.0 ± 1.3%), and Rib-12d group (5.5 ± 1.2%). This result suggested that BP tissues cross-linked by the Rib method obviously improved the resistance to elastase.

**FIGURE 7:**
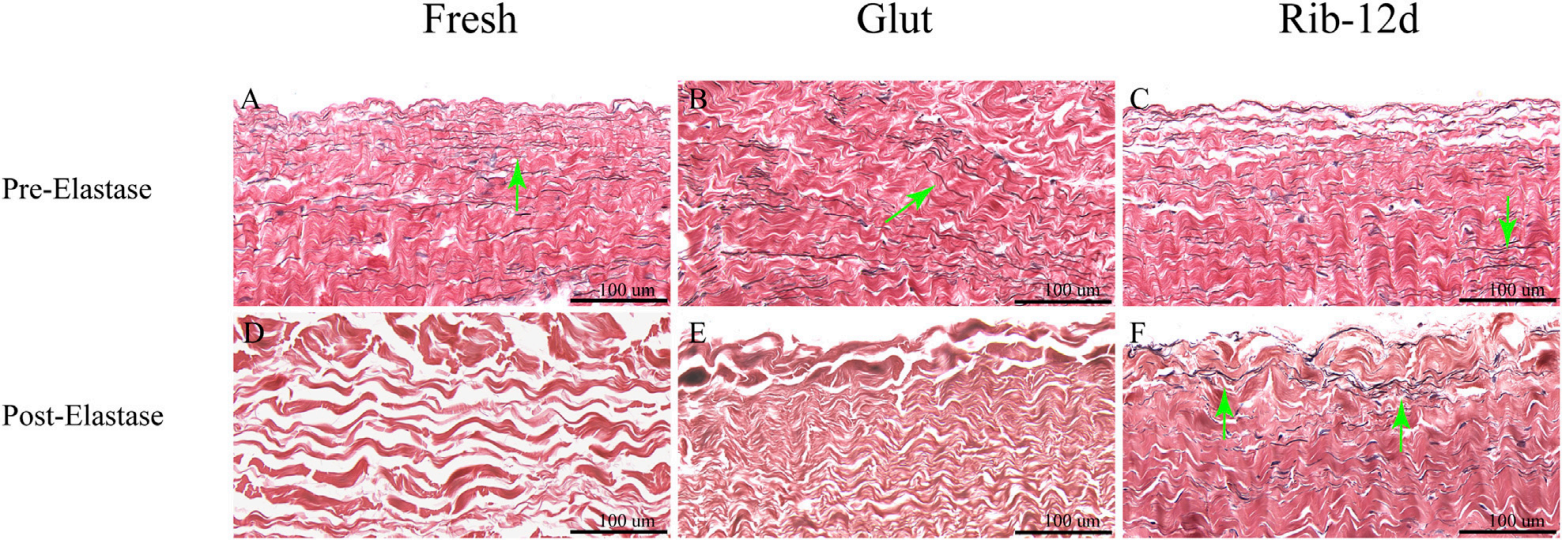
Resistance to the elastase enzymatic challenge of the BP with elastic van-Gieson (EVG) staining. **(A,D)** Fresh BP. **(B,E)** Glut-treated BP. **(C,F)** Rib-treated BP. Elastin are stained black strips (green arrow). Scale bars: 100 μm **(A–F)**.

BP tissues digested by elastase were embedded in paraffin again and stained with the EVG method to observe the elastin morphological structure. After the challenge with elastase, the BP structure of the fresh group was loose and no elastin was observed (Figure 7D). Although the structure was compact in the Glut group, no elastin was observed (Figure 7E). The Rib-12d group showed a large number of elastic fibers dyed in black stripes, and fibers were intact and contiguous in morphology (Figure 7F).

### Water Content

**FIGURE 8:**
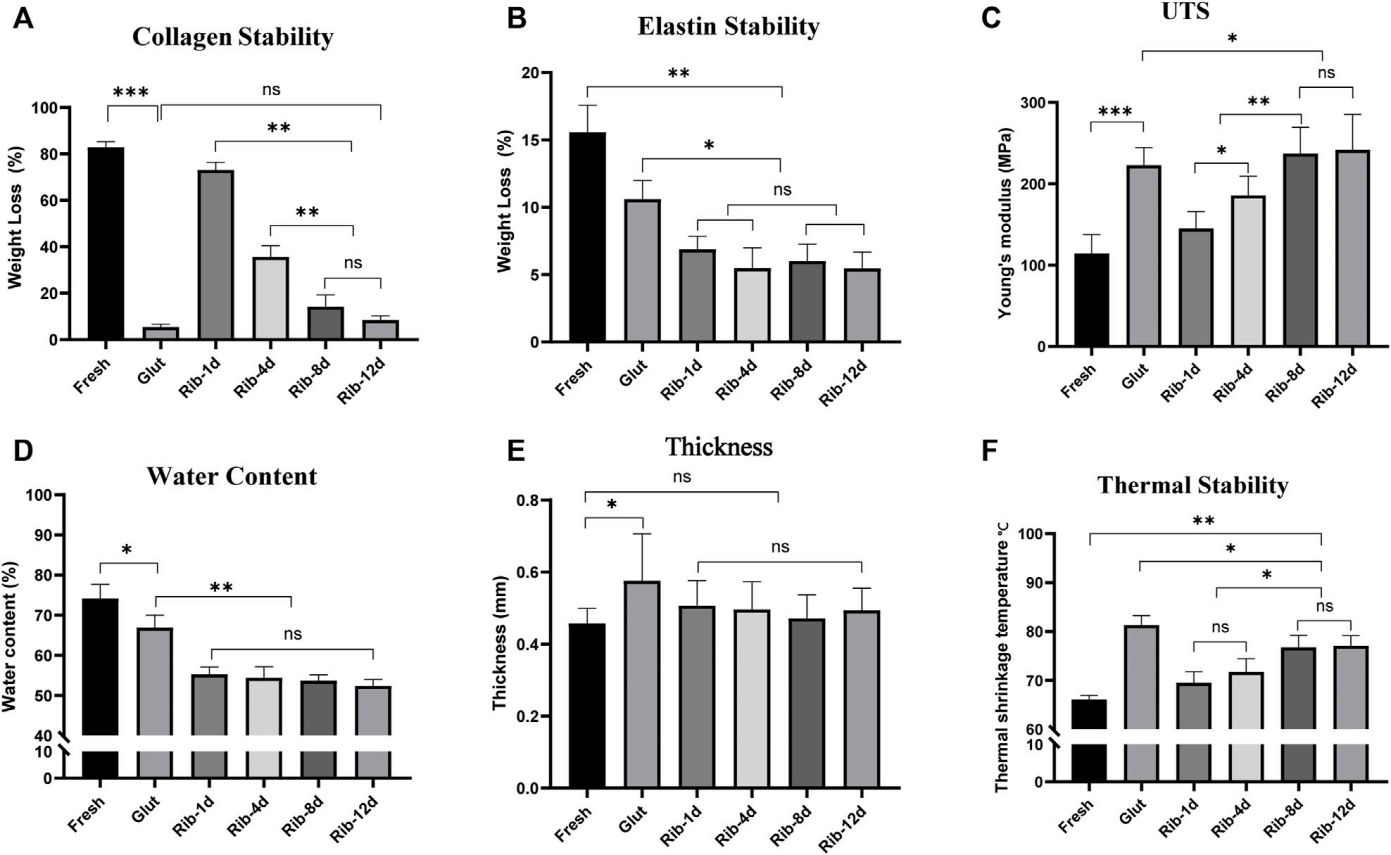
ECM stability of the BP *in vitro* study. **(A,B)** Relative weight loss of the BP (*n* = 6) after 24 h collagenase and 48 h elastase digestion. **(C)** Young’s modulus of the BP (*n* = 12) in uniaxial tensile testing. **(D)** Water content of the BP (*n* = 4). **(E)** Thickness of the BP (*n* = 12). **(F)** Thermal shrinkage temperature of the BP (*n* = 6) (*p < 0.05, **p < 0.01, and ***p < 0.001; ns represents no significant difference).

Compared with the fresh BP (74.2 ± 3.5%), the tissues had lower water content after crosslinking (*p* < 0.01, Figure 8D). Furthermore, the water content of Rib-treated BP (Rib-1d: 55.3 ± 1.8%, Rib-4d: 54.4 ± 2.7%, Rib-8d: 53.7 ± 1.5%, Rib-12d: 52.4 ± 1.6%) was significantly lower than Glut-treated BP (66.9 ± 3.1%) (*p* < 0.001).

### Biomechanics Stability Studies and Thickness

Uniaxial tensile testing was used to measure the biomechanical properties of BP tissues. Young’s modulus was calculated to reflect the stability of the material to mechanics (Figure 8C). The Rib-4d group (185.8 ± 23.6 MPa) was significantly higher than the fresh group (114.1 ± 23.4 MPa). A difference was found between the long-term group (Rib-12d: 241.9 ± 43.8 MPa) and the Glut group (222.7 ± 21.8 MPa). The maximum tensile stress of the fresh group (14.5 ± 5.1 MPa) was significantly lower than that of the Rib-12d group (23.6 ± 8.3 MPa) and Glut group (23.5 ± 9.8 MPa), and no difference was observed between the two groups (Table 1). The maximum load value of the BP in the Rib-12d group (117.8 ± 45.9 N) was significantly higher than that of the Fresh group (66.1 ± 22.1 N).

**TABLE 1:** Uniaxial tensile testing.

	Thickness (mm)	Max stress (MPa)	Max load(N)	Strain (%)	Young’s modulus (MPa)
Fresh	0.45 ± 0.04	14.5 ± 5.1	66.1 ± 22.1	21.6 ± 11.3	114.1 ± 23.4
Glut	0.57 ± 0.08	23.5 ± 9.8	140.0 ± 77.1	16.9 ± 7.4	145 ± 21.0
Rib-1d	0.51 ± 0.06	17.3 ± 6.8	86.6 ± 34.2	17.9 ± 4.6	222.7 ± 21.8
Rib-4d	0.49 ± 0.07	17.8 ± 5.2	89.9 ± 30.7	17.6 ± 10.1	185.8 ± 23.6
Rib-8d	0.47 ± 0.05	19.1 ± 3.5	91.9 ± 17.8	15.1 ± 4.2	237.2 ± 32.4
Rib-12d	0.49 ± 0.06	23.6 ± 8.3	117.8 ± 45.9	15.6 ± 5.4	241.9 ± 43.8

The strain of the fresh group was 21.6 ± 11.3%, which was higher than that of the Glut group (16.9 ± 7.4%) and Rib-1d group (17.9 ± 4.6%) (Table 1). Correspondingly, the thickness of the fresh group was 0.45 ± 0.04 mm, which was thinner than that of the Glut group (0.57 ± 0.08 mm) and Rib-1d group (0.51 ± 0.06 mm). Although the crosslinking process made the BP tissues thicker, it was inevitable that the Rib-12d group (0.49 ± 0.06) did not thicken as much as the Glut group (0.57 ± 0.08 mm) (Table 1).

### Thermal Stability Test

It has been demonstrated that T_d_ is related to the thermal stability of collagen in biomaterials. Td of the long-time group (Rib-8d: 76.8 ± 2.4°C, Rib-12d: 77.1 ± 2.1°C) was significantly higher than that of the fresh group and short-time group (Rib-1d: 69.5 ± 2.3°C). This result shows that the Rib crosslinking method can effectively improve the thermal stability of collagen in BP tissues, especially with longer crosslinking times (Figure 8F).

### Cytocompatibility

**FIGURE 9:**
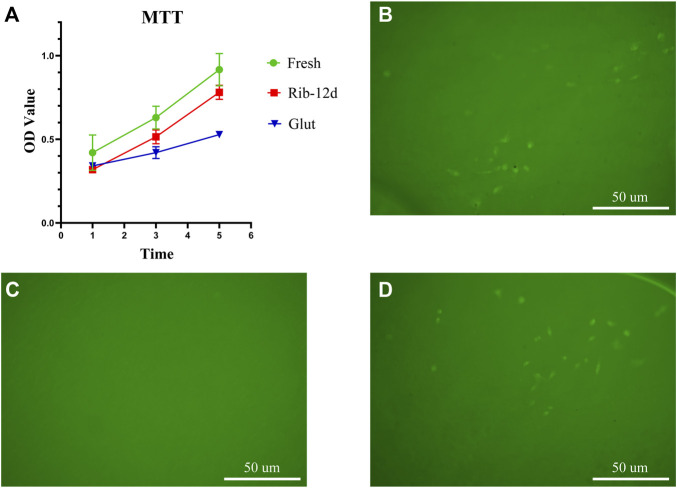
Cytocompatibility of the biomaterial. **(A)** Cell viability was tested by MTT assay at 1, 3, and 5 days (*n* = 3). **(B–D)** HUVEC growth on fresh BP, Glut-treated BP, and Rib-treated BP after 7 days of culture. Scale bars: 50 μm.

The cytotoxicity of the biomaterial was estimated by an MTT assay and EAhy926 growth test (Figure 9). The relative growth ratios (RGRs) of EAhy926 were determined in the presence of leach liquor from the tissues at concentrations after 1, 3, and 5 days of culture (Figure 9A). No apparent differences were observed between the groups on day 1 in the RGRs. On day 5 in the RGRs, there was an insignificant difference between the different groups.

EAhy926 growth on the surface of the biomaterial was evaluated by fluorescence imaging. After 7 days of culture, no visible cells could be observed on the surface of the Glut group (Figure 9C). In contrast, both the surface of the Rib and fresh patches were covered by EAhy926 with the normal morphological phenotype of endothelial cells (Figure 9 B, D). EAhy926 seeded on Rib-treated patches retained higher viability than EAhy926 seeded on Glut-treated patches.

### *In Vivo* Study

**FIGURE 10:**
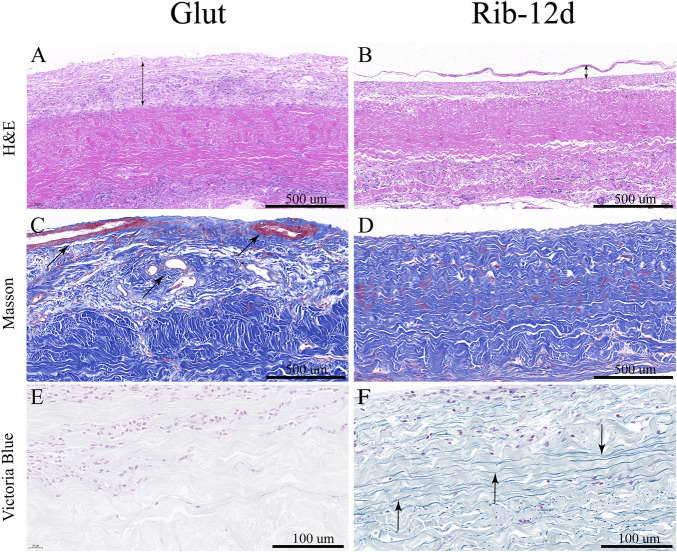
ECM integrity and histological staining after implanted into rats for 90 days. **(A,B)** H&E staining of the BP. Nuclei were stained purple, and the cytoplasm was stained pink. The thickness of the fibrous capsule (black double-arrow). **(C,D)** MT staining of the BP and the collagen are stained blue, and new capillary-like tubes are stained red (black arrow). **(E,F)** Victoria Blue staining of the BP. Elastin fibers were stained dark blue stripes (black arrow). Scale bars: 500 μm **(A–D)** and 100 μm **(E,F)**.

BP tissues cross-linked by the Glut and Rib method were implanted in rats to study the calcification tendency and integrity of the ECM. After 90 days, BP tissues were harvested and stained with the Victoria Blue method to observe elastin. The loss of elastin content in the Glut group was very obvious, and no elastin was stained (Figure 10E). ECM components such as elastin were preserved more firmly after implantation in the Rib-12d group. A large amount of elastin dyed dark blue stripes, and the fiber network was continuous and complete (Figure 10F). The specimens were stained by the MT method, and the collagen fibers of the two groups were basically intact (Figures 10C,D). More new capillary-like tubes were formed in the Glut group (Figure 10C). Red blood cells (RBCs) were diffused into BHV leaflets by the neovascularization procedure.

**FIGURE 11:**
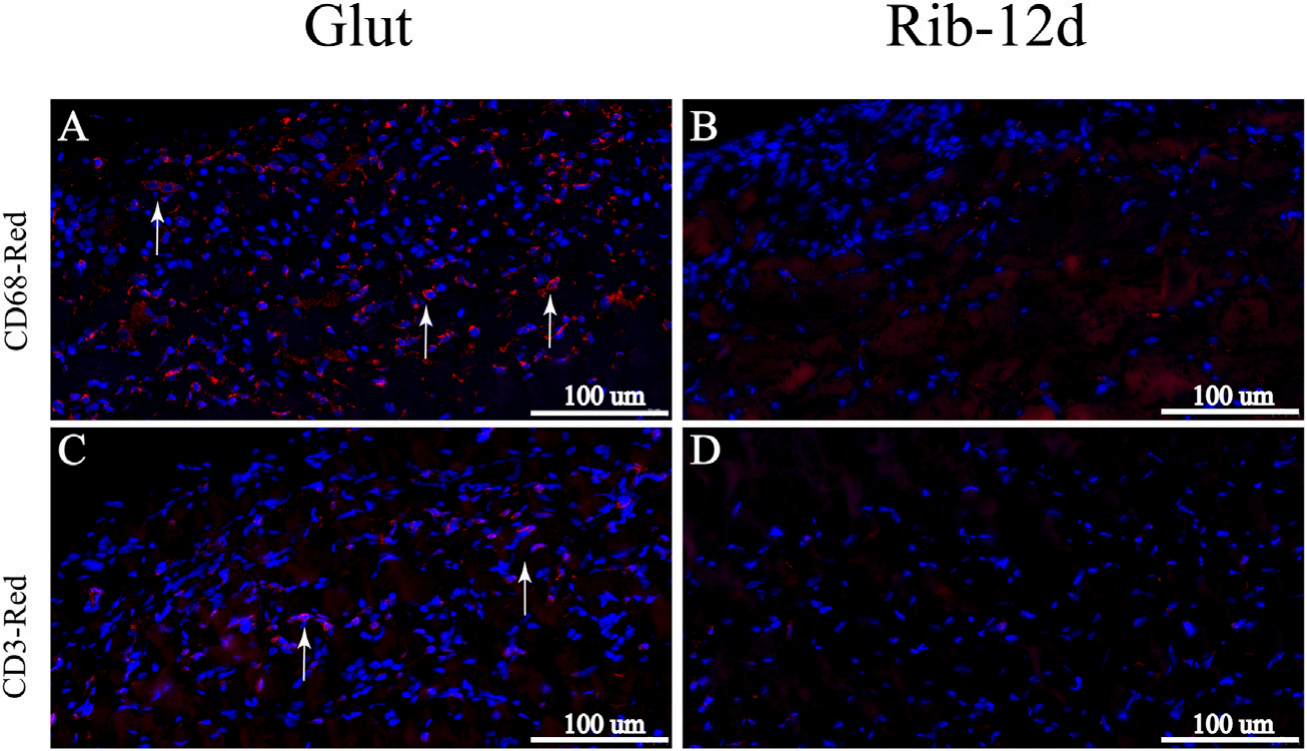
Immunological characterization of the inflammatory response of BP implanted into rats for 90 days. **(A,B)** IHC images. Macrophages were labeled with the CD68 marker and stained red (white arrow). **(C,D)** IHC images. T cells were labeled with the CD3 marker and stained red (white arrow). Scale bars: 100 μm **(A–D)**.

After implantation, H&E-stained images showed that the Glut group presented with abundant host inflammatory cellular activity and was surrounded by thicker fibrous capsule formation than the Rib group (Figure 10A). Immunofluorescence (IF) staining was used to analyze the phenotype of inflammatory cells that infiltrated around the implants. More CD68-marked macrophage cells were recruited around the BP in the Glut group after implantation than in the Rib-12d group (Figures 11A,B). Moreover, more CD3-marked T cells were recruited around the BP in the Glut group (Figures 11C,D). This finding is indicative of a severe chronic inflammatory response in the Glut group BP.

### Calcification

**FIGURE 12:**
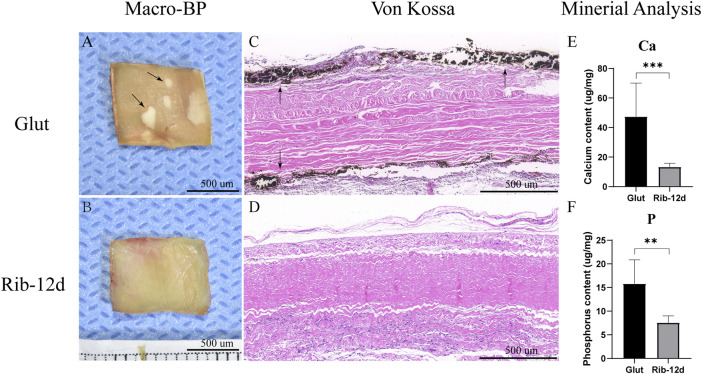
Calcification of the BP post-implantation into male juvenile rats for 90 days. **(A,B)** Macro image of the calcium nodules (black arrow). **(C,D)** von Kossa staining. Calcification deposits were stained black (black arrow). **(E,F)** ICP-7400 quantification of calcium and phosphorus contents in Glut- or Rib-treated BP (*n* = 12) (*p < 0.05, **p < 0.01, and ***p < 0.001) Scale bars: 500 µm.

Typical calcification could be found in the Glut group after implantation for 90 days. There were several white, hard, irregular calcium nodules on the surface of the Glut-BP in the macroscopic image (Figure 12A). The surface of the Rib-BP was smooth and flat (Figure 12B). Von Kossa staining on Glut-treated BP showed heavy, extensive calcification on both sides of the implants (Figure 12C). Rib-treated BP showed no calcification after implantation (Figure 12D). This was further demonstrated by quantitative mineral analyses with ICP-7400. We found that the Glut samples contained 47.5 ± 22.7 μg/mg of dry tissue calcium, and the Rib samples contained 13.4 ± 2.4 μg/mg (Figure 12E). Glut samples contained 15.8 ± 5.1 μg/mg of dry tissue phosphorus, and Rib samples contained 7.6 ± 1.4 μg/mg (Figure 12F). In the Glut group, both the calcium content and the phosphorus content were significantly higher than those in the Rib group.

## Discussion

### Glut Method—a Product of the Age

More than half a century has passed since Glut replaced formalin. Glut crosslinking technology has become the main method to prepare biomaterials and has been widely used in clinical practice (Lopez-Moya et al., 2018). However, this method inevitably has disadvantages during implantation. Biomaterials treated with Glut are prone to calcification because of the deposition of calcium–phosphate crystals, which leads to stiffness of the valve leaflets and dysfunction (Manji et al., 2006). Glut could crosslink the collagen component of the ECM but had no effect on elastin, and the degradation of elastin would accelerate the degeneration of the THV (Tam et al., 2015). Cytotoxic-free aldehyde groups were brought into the biomaterial tissue with the Glut method, which would induce the recognition and attack of the graft by the immune-inflammatory response, leading to the death of host cells and formation of cell debris (Shang et al., 2017). In summary, the durability of the THV was significantly reduced, especially failing faster in younger patients (Rajamannan et al., 2011; Mylotte et al., 2015; Kostyunin et al., 2020).

Therefore, we proposed a new crosslinking method in the preparation of the THV to pursue excellent anti-calcification ability, more comprehensive stability of the ECM, better cell compatibility, and mild immune inflammation.

### Rib Method—a Novel Crosslinking Technology

Krishnakumar et al. (2017b)evaluated different crosslinking agents on hybrid biomimetic collagen–hydroxyapatite composites for regenerative medicine including ribose. In our study, ribose was used to crosslink heart valve biomaterials and the possible mechanism of the reaction is shown in Figure 1A. Before the tissues were fixed, native BP had to be decellularized because cell debris might cause an immune response and calcification *in vivo* (Zhou et al., 2010). After the decellularization procedure, Rib was added to react with amine groups of collagen, which is the main component of the ECM (Krishnakumar et al., 2017b). The crosslinking of biomaterials was supposedly achieved through a general mechanism of the Amadori rearrangement (Figure 1A). First, the five-carbon ring of Rib was opened (Figure 1A-a), and Rib reacted with the amine groups of collagen to form enamine bonds (Figure 1A-b). The structure of enamine was not stable; it could easily transfer into alkenes (Figure 1A-c) and carbonyl groups (Figure 1A-d) through the Amadori rearrangement. The carbonyl group on the BP continued to attack the adjacent amine groups of another collagen fiber through nucleophilic addition (Figure 1A-e), finally producing new links between protein molecules.

^1^H-NMR spectroscopy and ninhydrin assays further demonstrated changes in the organic structure, and the crosslinking process was time-related (Figures 3A,B). The crosslinking degree mainly depends on the time of the reaction. The BP was soaked in the Rib solution for a long time and was fully reacted to form steady covalent bonds (Yamagishi, 2011; Liu et al., 2017). Liu et al. (2017)described the relationship between the color of the biomaterial and the degree of crosslinking. When the reaction time is longer, the degree of crosslinking is higher, which is manifested as the darker the color of the biomaterial (Figure 1B), the stronger the biomechanics properties (Figure 8C), and the higher the thermal shrinkage temperature (Figure 8F).

### More Compact Structure

During the TAVR operation, the biomaterial was compressed and folded within a delivery sheath (Mylotte et al., 2015). When the THV is crimped, the thickness of the tissues should be as thin as possible to pursue minimal invasion (Mylotte et al., 2015; Rodriguez-Gabella et al., 2017). The Glut crosslinking method thickens the tissues (Caballero et al., 2017), and the looser structure is more easily infiltrated by host cells and new capillaries (Morvan et al., 2019). The Rib group BP showed lower water content, thinner thickness, and a more compact fiber structure compared with the Glut group (Figures 4C,F, 8D,E). The probable explanation for the compact structure shown by the Rib group is that long-time crosslinking formed stable and irreversible network links between collagen (Krishnakumar et al., 2017a). The basic spatial orientation of collagen fibers determines the regular reticulate structures of biomaterials (Monte-Nieto et al., 2020).

### More Stable Extracellular Matrix

Collagen and elastin served as the backbone of the ECM. Collagen comprises a triple helix structure and the side chains are rich in functional amino groups (Gu et al., 2019). For example, genipin, epoxy compounds, and Glut could form stable covalent bond links within collagen fibers (Inostroza-Brito et al., 2017; Umashankar et al., 2017; Zhuravleva et al., 2021). Elastin mainly comprises alternately arranged hydrophobic amino acids and hydrophilic amino acids (Vindin et al., 2019). Polyphenols such as pentagalloyl glucose (PGG) and naringenin have been reported to fix elastin by forming hydrogen bonds (Isenburg et al., 2006). The collagen network provides the ability to resist chemical, biological, and mechanical challenges in the THV (Tam et al., 2017). Preserved elastin provides the ability to decrease calcification and increase tissue flexibility in the THV (Isenburg et al., 2006; Shavandi et al., 2019).

Through *in vitro* and *in vivo* studies, according to the enzyme treatment test and rat subcutaneous implantation test, we assessed the stability of ECM components (Raghavan et al., 2009). Although GLUT-treated BP showed stabilization of collagen fibers (Figure 6E) (Figure 10C), we found complete fragmentation of the elastin fibers by EVG staining and Victoria Blue staining (Figure 6E) (Figure 10E). In contrast, we found that Rib-treated BP had a clear protective effect on both elastin and collagen (Figure 7F) (Figure 10F), which was lacking in the Glut crosslinking method. Clinical research also indicates that elastin may not be preserved by the Glut crosslinking method in the body (Manji et al., 2014). This depletion of the ECM could be a large contributing factor to the structural degradation of Glut-treated BP (Monte-Nieto et al., 2020).

The *In vivo* and *in vitro* results are highly consistent. We have proven that the Rib crosslinking method can improve the stability of the ECM and effectively prevent the degradation of collagen and elastin, thereby increasing the durability of the THV.

### More Moderate Inflammatory Response

The remnant-free aldehyde group was brought into the biomaterial by the Glut crosslinking method (Manji et al., 2006). When Schiff base bonds were degraded, more toxic aldehyde groups were released. All of these factors could cause severe host cellular responses to implants (Shang et al., 2017). Innate and adaptive immune responses may lead to irreversible damage and calcification in the THV. The presence of macrophages on the interface of the biomaterial tissue suggested that the host immune response was persistent and inevitable in the Glut group BP (Dalgliesh et al., 2018). The *in vivo* study demonstrated that Glut group BP recruited more macrophages and T cells than Rib group BP (Figures 11A,C), which might trigger an attack from inflammatory cells and accelerate the degeneration of the THV.

On the other hand, the formation of a fibrosis capsule and new capillary infiltration around the biomaterial tissues were signs of chronic inflammation (Kostyunin et al., 2020). Under the influence of blood pressure, RBCs diffuse into THV leaflets through the capillary in the fibrosis capsule and can cause dystrophic calcification. The accumulation of RBCs was associated with capillary-like cavities in areas of tissue loosening and delamination (Morvan et al., 2019). The more compact structure and more moderate inflammatory response of the Rib group could explain the thinner fibrosis capsule and less new capillary infiltration than the Glut group after implantation for 90 days (Figures 10B,D).

### More Effective Reducing Calcification

Calcification is the main factor leading to reduced durability of the THV (Simionescu, 2004; Rajamannan et al., 2011; Rodriguez-Gabella et al., 2017). The fundamental mechanisms of calcification in biomaterials are still incompletely understood. Recent studies implicated that including the crosslinking method, ECM degradation, and immune-inflammatory response, all of these factors contributed to the process of calcification (Zilla et al., 2008; Kostyunin et al., 2020). Glut valves stained intense black with Von Kossa staining, indicative of severe calcification of the biomaterials. This deposition of calcification in the subcutaneous rat model corresponds to long-term clinical implantation (Brown et al., 2009). Compared with Glut-treated valves, no calcification was found in Von Kossa staining in Rib-treated valves, and the quantitative mineral analysis results were lower for both calcium and phosphorus (Figure 12). The Rib crosslinking method showed anti-calcification ability and the potential to improve the durability of the THV.

## Conclusion

A novel crosslinking method for THV fabrication, Rib treatment technology, has been demonstrated to produce a biomaterial that possesses a stable ECM and anti-calcification ability. The stability of the ECM was confirmed *in vitro* and *in vivo* studies. Excellent biocompatibility of the THV reduced the immune-inflammatory response and attack from host cells. Furthermore, the data suggested that long-time crosslinking biomaterials performed a compact structure and preserved elastin, which is especially suited for TAVR operation. Such creative development may improve the durability of the THV in the future.

## Data Availability

The original contributions presented in the study are included in the article/Supplementary Material; further inquiries can be directed to the corresponding author.
